# DOES PERSONALITY ALWAYS MATTER? EXAMINING THE MODERATING EFFECT OF AGE ON THE PERSONALITY-HEALTH LINK

**DOI:** 10.1093/geroni/igac059.1467

**Published:** 2022-12-20

**Authors:** Jing Luo, Bo Zhang, Eileen Graham, Daniel Mroczek

**Affiliations:** Northwestern University, Chicago, Illinois, United States; Texas A&M University, College Station, Texas, United States; Northwestern University, Chicago, Illinois, United States; Northwestern University, Chicago, Illinois, United States

## Abstract

The current research examined how the associations between the level and changes in the Big Five personality traits and different types of health outcomes (self-rated, physical, cognitive, and physiological health outcomes) differ across ages over the lifespan (Sample 1, age range: 15-100) and during the aging process (Sample 2, age range: 50-109) in particular. Using data from the two large longitudinal studies, we observed three important patterns based on the results. First, levels and changes in personality traits demonstrated substantial effects on health across different life phases, with the effects observed even in very old ages. Second, overall, the prospective relations between personality traits/changes in personality traits and health outcomes increased in strength in mid adulthood and/or early stages of late adulthood; however, the strength of their connections diminished in very old ages. Finally, there were some trait-specific and health outcome-specific patterns in the age-differential associations between personality and health.

